# Supplementary material for: Historical Wines of Portugal: The Classification, Consumer Associations and Marketing Implications
Source: Foods. 2021 Apr 29;10(5):979. doi: 10.3390/foods10050979 (PMC8146683; doi:10.3390/foods10050979)
Supplement: Supplementary file 1 [file foods-10-00979-s001.zip › foods-1138116-supplementary.pdf]

Table S1. Some examples of wines classified as Historical Wines of Portugal (photos courtesy of V. Loureiro).

Vinho Verde Enforcado

This wine has its roots in Etruscan (pre-Roman) viticulture, with its tree climbing vines bearing high similarity to the Married vine or *vite maritata* wine landscapes of Tuscany in the early 20th century. These wines are young, naturally fizzy (due to malolactic fermentation), low in ethanol and can be white or dark red, both from autochthonous grape varieties (mainly consumed locally to complement the richness of local gastronomy). Production yields are relatively high due to high plant vigor benefiting from intermediate crop (e.g., corn, beans, potatoes) fertilization and irrigation.

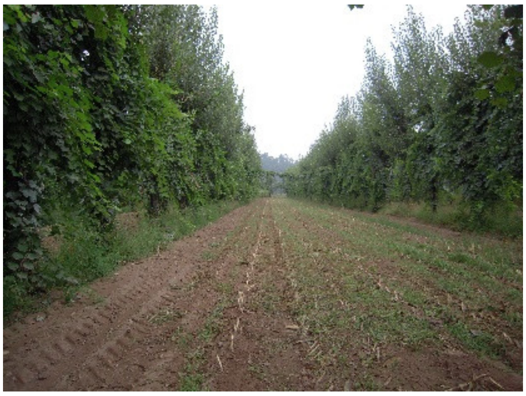

Palhete Medieval de Ourém

This is one of the few European wines still made according to the rules of the Cistercian monks from Burgundy, in the medieval ages, in an exclusively artisanal way. The wine is a claret produced in a warm climate in order to obtain the color of the "blood of Christ".
White grapes are fermented without skins and the wine is racked to wooden barrels until 80% capacity is reached. The barrels are filled with 20% juice and skins from red grapes, named *Tinta*, as dye, that had previously fermented together for a week. Wine in barrels is topped by a vase with red grapes that contact wine through a hole, thus preventing acetification. Ethanol concentration may attain 14% (*v/v*).

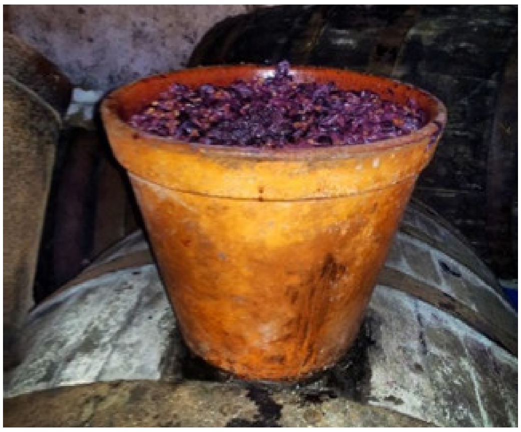

Vinho do Chão de Areia de Colares

This wine is made exclusively from grapes of ungrafted vines planted in clay soil and covered by more than two meters of sand, nearby the Atlantic Ocean shore. Crawling vines are protected from sea winds by palisades. The varieties are red (*Ramisco*) and white (*Malvasia de Colares*), which naturally protected from phylloxera.
Resulting wines have high quality and reputation, also due to their ability to age well. The production of these wines has all but disappeared under the threat of urban development brought about by the growth of the Lisbon neighborhood.

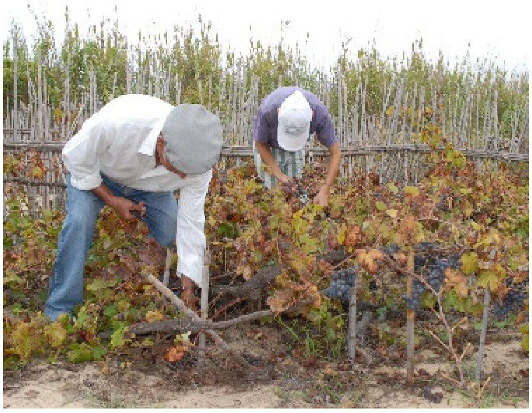

Vinho do Pico

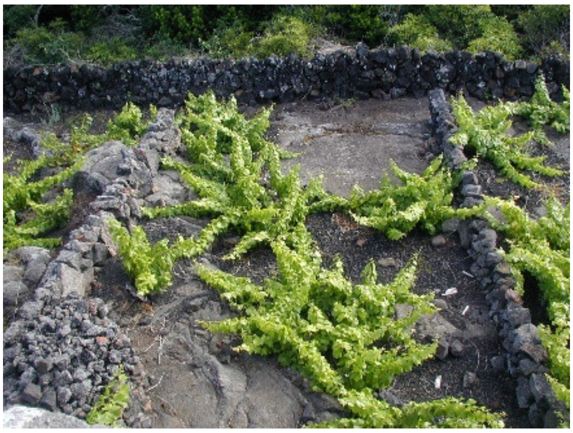

This dry white wine and other local fortified white wines result from artisanal forms of production (vine and wine) brought by the first settlers to the Azores archipelago, and still remain active today on some of the islands. Vines are planted on basalt rock fissures and protected from the sea wind by basalt stone walls. They represent a perfect adaptation of the *Verdelho* white varietal to a *terroir* dominated by the islands' position in the middle of the Atlantic Ocean and their volcanic origin [4]. Importantly, the uniqueness and ancient character of such *terroir* in the island of *Pico* has been awarded world heritage status by UNESCO.

Vinho da Talha

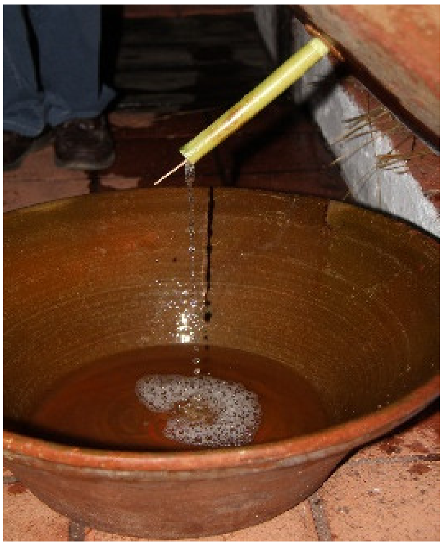

These wines were introduced by the Romans in the south of Portugal (Alentejo region) around 2000 years ago. To make these wines, crushed grapes are placed inside large clay jar vats (ca. 1000 L) to ferment spontaneously [5]. During this process, grape skins rise to the surface and form a solid layer, which is stirred manually with a wooden stick and forced to dive back down into the must in order to transmit the wine more color, aroma and flavor. After fermentation is completed, these masses settle at the bottom of the vat. On its wall, near the base, there is a hole where a filtering faucet is placed. The wine crosses the filter formed by the masses of grapes, which come out pure and limpid and ready to drink. Similar ancient winemaking methods have been observed in rural parts of Spain with origins that can be traced back to South Caucasus [6]. *Vinho da Talha* is traditionally made with local white varieties. Local red grapes may also be used.

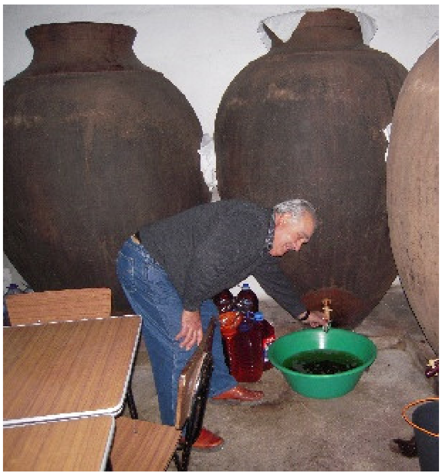

Vinho Petroleiro

This is a wine typical from *Vila de Frades* (Friars' village) differing from *Vinho de Talha* concerning grape composition. In the vineyard 20% of red varieties are planted among the remaining white grapes. The clay jars are filled with the mixture of grapes harvested together. The wine has a petrol-like color, originally intended to mimic the "blood of Christ" in a warm climate region.

Table S2. Frequency distributions of questionnaire respondents' classes of familiarity with grape varieties (*n* = 641).

Wine knowledge	Baga (%)	Castelão (%)	Pinot Noir (%)	Shiraz (%)
1 = Never heard of it	44.9	26.1	29.6	33.5
2 = Have heard of it, but cannot recall ever drinking wine made from it	17.3	15.8	22.8	9.0
3 = Have heard of it and can recall drinking wine made from it at least once	11.7	18.9	20.3	12.2
4 = Have heard of it and can recall drinking wine made from it several times	26.1	39.3	27.3	45.2

Table S3. Factor analysis of the distribution channels where questionnaire respondents (n=641) expected to find a wine classified as HWP for sale.

Dimensions[1]	Loadings
Specialty stores	
Eigen value = 2.726; Explained variance = 38.9%; Mean ± SD = 4.60 ± 1.18	
Wine clubs	0.90
Wine stores	0.89
Wineries	0.69
Online stores	0.66
General retail	
Eigen value = 1.994; Explained variance = 28.5%; Mean ± SD = 3.04 ± 1.31	
Supermarkets	0.95
Hypermarkets	0.90
Neighborhood grocery stores	0.64

[1]Rated from 1 = "Never" to 6 = "Always".

Table S4. Factor analysis of the locations where questionnaire respondents (*n* = 641) expected to drink a wine classified as HWP.

Dimensions[1]	Loadings
Wineries and wine events	
Eigen value = 3.985; Explained variance = 44.3%; Mean ± SD = 3.88 ± 1.26	
Wine tours or winery visits	0.86
Events held by national or local authorities	0.79
Wine fairs, festivals or similar events	0.67
Home	
Eigen value=1.334; Explained variance=14.8%; Mean ± SD = 3.97 ± 1.30	
At home	0.84
At the home of relatives of friends	0.82
HORECA	
Eigen value = 0.948; Explained variance = 10.5%; Mean ± SD = 3.22 ± 1.04	
Restaurants	0.81
Hotels and similar establishments	0.60
Ceremonies or special events	0.40
Wine bars or taverns	0.37

[1]Rated from 1 = "Never" to 6 = "Always".
